# Inflammatory (B) symptoms are independent predictors of myelosuppression from chemotherapy in Non-Hodgkin Lymphoma (NHL) patients – analysis of data from a British National Lymphoma Investigation phase III trial comparing CHOP to PMitCEBO

**DOI:** 10.1186/1471-2407-9-153

**Published:** 2009-05-18

**Authors:** Rohini Sharma, David Cunningham, Paul Smith, Graham Robertson, Owen Dent, Stephen J Clarke

**Affiliations:** 1 Imperial College, London, UK; 2 Royal Marsden Hospital, Sutton, Surrey, UK; 3 Lymphoma Trials Office, Cancer Research UK & UCL Cancer Trials Centre, 90 Tottenham Court Road, London, UK; 4 The University of Sydney, Department of Medicine, Concord Hospital, Concord, NSW, Australia

## Abstract

**Background:**

Toxicity from chemotherapy is highly variable, unpredictable and results in substantial morbidity and increased healthcare costs. New predictors of toxicity are required to improve the safety and efficacy of chemotherapy. Inflammatory or B symptoms in lymphoma are associated with elevated plasma inflammatory markers and predict worse treatment response and survival. Recent data suggest that systemic inflammation results in reduced hepatic drug metabolism and increased toxicity from chemotherapy. We investigated whether B symptoms were associated with greater toxicity in patients treated for non-Hodgkin lymphoma (NHL).

**Methods:**

The British National Lymphoma Investigation compared two chemotherapy regimens in older patients with aggressive NHL. Approximately 50% of patients had B symptoms. Demographic and toxicity data on 664 patients were analysed to identify predictors of toxicity by multivariate analysis, with particular reference to B symptoms.

**Results:**

Using univariate analyses, severe (grades 3–4) leucopenia, anaemia, thrombocytopenia, nausea and vomiting and diarrhoea occurred more frequently in patients with B symptoms. The associations between B symptoms and severe leucopenia (OR 1.7, p = 0.005) and anaemia (OR 2.3, p = 0.025) persisted after adjustment for other prognostic factors in multivariate analyses. The use of granulocyte colony stimulating factor reduced neutropenia in patients with both A and B symptoms.

**Conclusion:**

For the first time and in a large NHL cohort we have shown that inflammatory symptoms are independent predictors for myelosuppression from chemotherapy. These data will enable improved prognostication for toxicity and provide individualisation of therapy in NHL and other tumours. These findings also create the potential for strategies used prior to chemotherapy aimed at reducing systemic inflammation in order to improve drug metabolism and reduce treatment-related toxicity.

**Trial registration number:**

ISRCTN98741793

## Background

Cancer chemotherapy produces variable and unpredictable toxicities, which can cause significant morbidity, occasional mortality and result in substantial healthcare costs due to increased requirement for toxicity-related hospitalisation [1-3]. Non-Hodgkin lymphoma (NHL) is the fifth most common cancer by incidence in both men and women in the developed world [4] and is usually treated with combination chemotherapy. Although regarded as a chemotherapy-sensitive disease, over 50% of patients with the diffuse large B cell variant will die of their disease. The effective use of cancer chemotherapy is a balance between adequate anti-tumour effect and manageable normal tissue toxicities. There is evidence that dose reduction and delay for toxicity in NHL results in inferior response rates and survival than when dose intensity is maintained [5]. An improved ability to predict and/or prevent toxicity would substantially improve outcomes in NHL, and other malignancies.

The presence or absence of inflammatory or B symptoms (fever > 38°C, weight loss > 5% or night sweats) is an established negative prognostic factor in patients with NHL. B symptoms are associated with increased plasma levels of inflammatory proteins including C-reactive protein (CRP) [6] and cytokines such as interleukin-6 (IL-6) [7]. Elevated inflammatory proteins have been shown to correlate with other prognostic markers in NHL including ECOG performance status, β_2_-microglobulin levels and International Prognostic Index (IPI) [7,8]. In addition, NHL patients with elevated plasma inflammatory markers have lower response rates to chemotherapy and worse survival than those with normal levels [7-10].

The impact of inflammation on toxicity in NHL has not been extensively investigated. Chemotherapy induced toxicity is particularly relevant in NHL as many of the patients are older and relatively frail, and toxicities may lead to treatment interruption, dose reduction, major morbidity necessitating hospitalization, and even treatment-related death. There is evidence that loss of relative dose intensity in NHL can compromise treatment outcomes. For example, Kwak and colleagues reported that NHL patients who received > 75% of planned doxorubicin doses had markedly superior survival to those receiving lesser doses [5]. Improved dose individualization and avoidance of or reduction in the severity of toxicity would assist in maintaining dose intensity.

There is increasing evidence that a systemic inflammatory response occurs frequently in patients with malignancy, and is generally associated with worse clinical outcomes (reviewed in [11]). Furthermore, the presence of raised pro-inflammatory cytokines, including IL-6, has been shown to negatively impact on hepatic drug metabolism (reviewed in [12] and [13]). This concept is supported by data which demonstrated that reductions in CYP3A4 activity in patients with advanced cancer were correlated with increased plasma concentrations of IL-6 and CRP [14]. This was associated with reduced clearance and increased toxicity from docetaxel, a well-characterized substrate for CYP3A4 [14,15]. Most cancer drugs are metabolized by CYP3A4, including those used to treat NHL.

These data suggested the hypothesis that cancer patients with evidence of a tumour-induced inflammatory response would experience greater chemotherapy-related toxicity and worse treatment outcomes than patients without such an inflammatory response. An obvious circumstance in which to test such an hypothesis is NHL, due to the frequent presence and documentation of inflammatory symptoms, and because reduced dose intensity has an adverse prognostic impact in this condition. It is also timely to evaluate such a relationship as a number of anti-inflammatory treatments have been developed recently, including monoclonal antibodies to cytokines and cytokine receptors. These treatments could potentially be used to reverse impaired cytotoxic drug metabolism prior to commencement of chemotherapy.

A British National Lymphoma Investigation (BNLI) phase III study compared the efficacy and safety of two chemotherapy regimens (cyclophosphamide, doxorubicin, vincristine and prednisone (CHOP) or mitoxantrone, cyclophosphamide, etoposide, vincristine, bleomycin and prednisolone (PMitCEBO)) with or without granulocyte colony stimulating factor (G-CSF) in patients aged over 60 years with aggressive NHL [16]. This trial enrolled 784 patients of whom approximately 50% per arm had B symptoms. In the current study we have reanalysed these data to determine the prognostic impact of B symptoms on toxicity.

## Methods

The study procedures have been published previously [16]. Briefly, consenting patients over 60 years of age diagnosed with diffuse large B cell lymphoma who had adequate organ function and performance status were randomised in a two by two fashion to receive chemotherapy with either CHOP or PMitCEBO with or without G-CSF. The study was approved by the human research ethics committees of all participating centres.

### Prediction of toxicity

Toxicity was recorded during all cycles of treatment in regard to the worst grade of anaemia, leucopenia and thrombocytopenia and the symptoms of nausea/vomiting, diarrhoea, alopecia, mucositis, neuropathy and skin rashes according to standard criteria (National Cancer Institute Common Toxicity Criteria Version 3 (NCICTC v 3)-https://webapps.ctep.nci.nih.gov/webobjs/ctc/webhelp/welcome_to_ctcae.htm).

Several prognostic variables were evaluated for their impact on toxicity. These included age, sex, stage, performance status, plasma lactate dehydrogenase concentration (LDH), type of chemotherapy used, presence of B symptoms, presence of bone marrow involvement on either aspirate or trephine, and use of G-CSF. The International Prognostic Index was not used in analyses because it is a function of age, stage, performance status and LDH, all of which were included separately in multivariate modelling.

For some variables data were unavailable on moderately large proportions of the original total of 784 patients; for example 12.4% for LDH, 15.1% for bone marrow trephine and 16.7% for bone marrow aspirate. Initially each variable was checked against sex, age, disease stage, performance status, type of chemotherapy and whether or not GCSF was given to determine whether there were any systematic differences in the distribution of missing data, but none was found. In addition, other variables were compared with the haematological toxicity measures in the same manner. The only association found was that patients with data missing on bone marrow aspirate and trephine also tended to have data missing on haematological toxicity. This may have arisen because of matters of policy or availability of these tests in some centres from which patients were drawn.

Patients in this study were all aged 60 years or older. Age was dichotomized at 75 years because this is approximately the point at which normal mortality begins to take effect in modern Western societies.

The statistical significance of differences in contingency tables was assessed by the chi-squared test or Fisher's exact test. Odds ratios (OR) for contingency tables and their 95 per cent confidence intervals (CI) were calculated by logistic regression and their statistical significance was assessed by the Wald test. The level for statistical significance was p < 0.05. In multivariate modelling by logistic regression, all predictor variables having an association with an outcome variable with p < 0.1 were entered into an initial model which was then reduced by successive elimination of variables with p > 0.05. The provisional final model thus obtained was further examined by re-entering/removing every previously excluded variable separately until a model containing only variables with p < 0.05 resulted. Potential interactions between B symptoms and other variables were evaluated but no such effects were identified. Potential interactions among other variables were also explored but none having any material influence on the chosen final regression models were identified. As an additional check regarding missing data, for each final regression model, all patients who were excluded because of missing data on any variable in the model were compared with those who were included in relation to sex, age, disease stage, performance status, type of chemotherapy and whether or not GCSF was given, but no significant differences were found. It was concluded that the varying sub-sets of patients analysed were not unrepresentative of the total pool of 784 patients. Analyses were performed using SPSS Version 16 (SPSS Australasia Pty Ltd).

## Results

Of the 784 patients in the original study the following were excluded sequentially: four with no information on stage of disease, 20 with no information on B symptoms, and 96 with no information on one or more of the haematological toxicity measures (anaemia, leucopenia, thrombocytopenia), leaving 664 patients for analysis, most of whom had complete data on most variables analysed. The age and sex of patients, treatment groups, clinical characteristics at randomization, maximum haematological toxicity grades attained during treatment and frequency of toxicity symptoms are shown in Tables 1 and 2. The presence of B symptoms was associated with disease stages 3 or 4, performance levels 2–4, elevated LDH and positive bone marrow assessments (Table 3). In subsequent analyses patient age, disease stage, performance status and the toxicity variables were dichotomized as shown in Table 4.

**Table 1:** Distribution of patients by baseline clinical variables.

Variable	Category	Number (%)
		N = 664
Sex	Female	311 (46.8)
	Male	353 (53.2)
		
Age	Less than 75 years	496 (74.7)
	75 years or older	168 (25.3)
		
Chemotherapy	CHOP	334 (50.3)
	PMitCEBO	330 (49.7)
		
GCSF	No	330 (49.7)
	Yes	334 (50.3)
		
Stage	I	70 (10.5)
	II	176 (26.5)
	III	193 (29.1)
	IV	225 (33.9)
		
B symptoms	No	311 (46.8)
	Yes	353 (53.2)
		
ECOG Performance status	0	206 (31.0)
	1	264 (39.8)
	2	131 (19.7)
	3	39 (5.9)
	4	13 (2.0)
	No data	11 (1.7)
		
Plasma LDH concentration (mU/L)		
	Normal (0.2–0.5)	216 (32.5)
	Elevated (> 0.5)	381 (57.4)
	No data	67 (10.1)
		
Bone marrow aspirate	Not involved	508 (76.5)
	Involved	57 (8.6)
	No data	99 (14.9)
		
Bone marrow trephine	Not involved	487 (73.3)
	Involved	89 (13.4)
	No data	88 (13.3)

**Table 2:** Overall toxicity from chemotherapy in selected cohort.

Variable	Category	Number (%)
		N = 664
Anaemia stage	0	116 (17.5)
	1	241 (36.3)
	2	259 (39.0)
	3	39 (5.9)
	4	9 (1.4)
		
Leucopenia stage	0	171 (25.8)
	1	70 (10.5)
	2	96 (14.5)
	3	154 (23.2)
	4	173 (26.1)
		
Thrombocytopenia	0	503 (75.8)
	1	51 (7.7)
	2	45 (6.8)
	3	33 (5.0)
	4	32 (4.8)
		
Mucositis	0	347 (52.3)
	1	137 (20.6)
	2	98 (14.8)
	3	17 (2.6)
	4	3 (0.5)
	No data	62 (9.3)
		
Nausea/vomiting	0	329 (49.5)
	1	168 (25.3)
	2	68 (10.2)
	3	39 (5.9)
	4	5 (0.8)
	No data	55 (8.3)
		
Diarrhoea	0	434 (65.4)
	1	74 (11.1)
	2	50 (7.5)
	3	45 (6.8)
	4	7 (1.1)
	No data	54 (8.1)
		
Neuropathy	0	318 (47.9)
	1	192 (28.9)
	2	77 (11.6)
	3	26 (3.9)
	4	0
	No data	51 (7.7)
		
Skin rash	0	521 (78.5)
	1	50 (7.5)
	2	27 (4.1)
	3	7 (1.1)
	4	2 (0.3)
	No data	57 (8.6)

**Table 3:** Association between B symptoms and clinical variables.

Variable	Category	n	B symptoms present	p
Sex	Female	311	169 (54.3)	0.577
	Male	353	184 (52.1)	
				
Age	< 75 years	496	261 (52.6)	0.631
	GE 75 years	168	92 (54.8)	
				
Stage	Stage 0–2	246	94 (38.2)	< 0.001
	Stage 3–4	418	259 (62.0)	
				
Performance status	Level 0–1	470	210 (44.7)	< 0.001
	Level 2–4	183	137 (74.9)	
				
LDH	Normal	216	95 (44.0)	< 0.001
	Elevated	381	223 (58.5)	
				
Bone marrow aspirate	Negative	508	266 (52.4)	0.040
	Positive	57	38 (66.7)	
				
Bone marrow trephine	Negative	487	246 (50.5)	< 0.001
	Positive	89	62 (69.7)	

**Table 4:** Patients with grade 3 or 4 anaemia, leucopenia or thrombocytopenia by demographic, treatment and clinical variables. Number (per cent).

Variable	Category	n	Grade 3/4 leucopenia	p	Grade 3/4 anaemia	p	Grade 3/4 thrombocytopenia	p
B symptoms	No	311	124 (39.9)	< 0.001	14 (4.5)	0.011	17 (5.5)	< 0.001
	Yes	353	203 (57.5)		34 (9.6)		48 (13.6)	
								
Sex	Female	311	159 (51.1)	0.363	25 (8.0)	0.450	33 (10.6)	0.504
	Male	353	168 (47.6)		23 (6.5)		32 (9.1)	
								
Age	< 75 years	496	226 (45.6)	0.001	33 (6.7)	0.324	40 (8.1)	0.010
	≥ 75 years	168	101 (60.1)		15 (8.9)		25 (14.9)	
								
Chemotherapy	CHOP	334	129 (38.6)	< 0.001	27 (8.1)	0.392	40 (12.0)	0.056
	PMitCEBO	330	198 (60.0)		21 (6.4)		25 (7.6)	
								
GCSF	No	330	184 (55.8)	< 0.001	14 (4.2)	0.003	25 (7.6)	0.056
	Yes	334	143 (42.8)		34 (10.2)		40 (12.0)	
								
Stage	Stage 0–2	246	99 (40.2)	< 0.001	14 (5.7)	0.240	11 (4.5)	< 0.001
	Stage 3–4	418	228 (54.5)		34 (8.1)		54 (12.9)	
								
Performance status	Level 0–1	470	197 (41.9)	< 0.001	27 (5.7)	0.012	25 (5.3)	< 0.001
	Level 2–4	183	125 (68.3)		21 (11.5)		39 (21.3)	
								
LDH (mU/L)	Normal	216	96 (44.4)	0.038	7 (3.2)	0.008	5 (2.3)	< 0.001
	Elevated	381	203 (53.3)		34 (8.9)		52 (13.6)	
								
Bone marrow aspirate	Negative	508	248 (48.8)	0.005	31 (6.1)	0.046*	45 (8.9)	0.004
	Positive	57	39 (68.4)		8 (14.0)		12 (21.1)	
								
Bone marrow	Negative	487	228 (46.8)	0.001	30 (6.2)	0.083	40 (8.2)	0.001
Trephine	Positive	89	58 (65.2		10 (11.2)		18 (20.2)	

* Fisher exact p

### Leucopenia

In univariate analyses grade 3 or 4 leucopenia was significantly associated with the presence of B symptoms occurring in approximately 40% of patients without inflammatory symptoms compared with 58% of those with B symptoms (p < 0.001). Severe leucopenia also occurred more frequently in patients aged 75 years or older, having received PMitCEBO, having received GCSF, disease stage 3 or 4, performance status level 2 or higher, elevated LDH, positive bone marrow aspirate and positive bone marrow trephine (Table 4). There was no association with sex. The association between B symptoms and leucopenia persisted (OR 1.7, 95% CI 1.2–2.4) in multivariate analysis after adjustment for age, performance status, bone marrow involvement, type of chemotherapy and receipt of GCSF, the latter having a protective effect (Table 5).

**Table 5:** Logistic regressions for association between leucopenia, anaemia and thrombocytopenia and B symptoms and other potential predictor variables.

	Univariate OR^#^	p	Multivariate OR^#^	p
	(95% CI^@^)		(95% CI^@^)	
Leucopenia *				
B symptoms present	2.0 (1.5–2.8)	< 0.001	1.7 (1.2–2.4)	0.005
Performance status level 2–4	3.0 (2.1–4.3)	< 0.001	2.2 (1.5–3.3)	< 0.001
Bone marrow aspirate positive	2.3 (1.3–4.1)	0.006	2.2 (1.2–4.0)	0.015
Age 75 years or older	1.8 (1.3–2.6)	0.001	2.1 (1.4–3.1)	< 0.001
PMitCEBO given	2.4 (1.7–3.3)	< 0.001	2.1 (1.5–3.0)	< 0.001
GCSF given	0.6 (0.4–0.8)	0.001	0.5 (0.4–0.8)	0.001
Bone marrow trephine positive	2.1 (1.3–3.4)	0.002	-	
Stage 3 or 4	1.8 (1.3–2.5)	< 0.001	-	
LDH elevated	1.4 (1.02–2.0)	0.038	-	
				
Anaemia **				
B symptoms present	2.3 (1.2–4.3)	0.013	2.3 (1.1–4.7)	0.025
LDH elevated	2.9 (1.3–6.7)	0.011	2.6 (1.1–6.1)	< 0.001
GCSF given	2.6 (1.3–4.9)	0.004	2.3 (1.1–4.5)	0.020
Bone marrow aspirate positive	2.5 (1.1–5.8)	0.030	-	
Performance status level 2–4	2.1 (1.2–3.9)	0.013	-	
Bone marrow trephine positive	1.9 (0.9–4.1)	0.088	-	
				
Thrombocytopenia ***				
B symptoms present	2.7 (1.5–4.8)	0.001	-	
Performance status level 2–4	4.8 (2.8–8.2)	< 0.001	4.8 (2.5–9.2)	< 0.001
LDH elevated	6.7 (2.6–17.0)	< 0.001	4.0 (1.5–10.4)	0.005
Bone marrow trephine positive	2.8 (1.5–5.2)	0.001	2.9 (1.4–5.8)	0.003
PMitCEBO given	0.6 (0.4–1.0)	0.058	0.5 (0.3–0.9)	0.032
Stage 3 or 4	3.2 (1.6–6.2)	0.001	-	
Bone marrow aspirate positive	2.7 (1.4–5.6)	0.005	-	
Age 75 years or older	2.0 (1.2–3.4)	0.011	-	
GCSF given	1.7 (1.0–2.8)	0.058	-	

* The multivariate model is for 560 patients with complete data on all variables included in it.

** The multivariate model is for 597 patients with complete data on all variables included in it.

*** The multivariate model is for 522 patients with complete data on all variables included in it.

# Odds ratio

@ Confidence interval

### Anaemia

In univariate analyses grade 3 or 4 anaemia was significantly associated with the presence of B symptoms occurring in 10% of patients with B symptoms compared with 5% without (p = 0.011). Severe anaemia was also associated with diminished performance status, elevated LDH and a positive bone marrow aspirate (Table 4). Patients who had received GCSF were also more likely to experience grade 3 or 4 anaemia. There were no statistically significant associations with the other variables considered. The association between B symptoms and anaemia persisted in a reduced logistic regression model after adjustment for receipt of GCSF and elevated LDH (OR 2.3, 95% CI 1.1–4.7 – Table 5).

### Thrombocytopenia

Grade 3 or 4 thrombocytopenia was associated with the presence of B symptoms in univariate analysis and occurred in 6% and 14% of patients with A and B symptoms, respectively (p < 0.001, Table 4). Other features associated with thrombocytopenia were age 75 or older, stage 3 or 4 disease, level 3 or 4 performance status, elevated LDH, positive bone marrow aspirate and positive bone marrow trephine. There were marginally non-significant associations with type of chemotherapy and receipt of GCSF, but no association with sex. In multivariate analysis, however, the significant association between B symptoms and thrombocytopenia was not sustained after adjustment for performance status, LDH, bone marrow trephine and type of chemotherapy (Table 5).

### Non-haematological toxicity

There were no associations between B symptoms and mucositis, neuropathy or skin rashes. In univariate analyses moderate to very severe (grades 2–4) nausea and vomiting (21% vs 15%) and diarrhoea (21% vs 12%) were significantly more common among patients with B symptoms (Table 6). However the associations between B symptoms and nausea and vomiting and diarrhoea disappeared after adjustment for other variables independently associated with these symptoms (Table 7).

**Table 6:** Patients with moderate to very severe nausea/vomiting or diarrhoea by demographic, treatment and clinical variables.

Variable	Category	Nausea/vomiting	p	Diarrhoea	p
B symptoms	No	43/285 (15.1)	0.048	34/284 (12.0)	0.003
	Yes	69/324 (21.3)		68/326 (20.9)	
					
Sex	Female	68/285 (23.9)	0.001	54/286 (18.9)	0.179
	Male	44/324 (13.6)		48/324 (14.8)	
					
Age	< 75 years	84/453 (18.5)	0.869	65/452 (14.4)	0.009
	GE 75 years	28/156 (17.9)		37/158 (23.4)	
					
Chemotherapy	CHOP	72/315 (22.9)	0.003	58/313 (18.5)	0.219
	PMitCEBO	40/294 (13.6)		44/297 (14.8)	
					
Stage	Stage 0–2	40/227 (17.6)	0.705	30/225 (13.3)	0.086
	Stage 3–4	72/382 (18.8)		72/385 (18.7)	
					
Performance status	Level 0–1	60/430 (14.0)	< 0.001	50/429 (11.7)	< 0.001
	Level 2–4	48/169 (28.4)		50/171 (29.2)	

Number (per cent).

**Table 7:** Logistic regression for association between moderate to very severe nausea and vomiting and diarrhoea and B symptoms and other potential predictor variables.

	Univariate OR^#^ (95% CI^@^)	p	Multivariate OR^#^ (95% CI^@^)	p
Nausea and vomiting*				
B symptoms present	1.5 (1.0–2.3)	0.049	-	
Performance status level 2–4	2.4 (1.6–3.8)	< 0.001	2.6 (1.6–4.0)	< 0.001
Male sex	0.5 (0.3–0.8)	0.001	0.5 (0.3–0.7)	0.001
PMitCEBO given	0.5 (0.3–0.8)	0.004	0.5 (0.3–0.8)	0.003
				
Diarrhoea**				
B symptoms present	1.9 (1.2–3.0)	0.004	-	
Performance status level 2–4	3.1 (2.0–4.9)	< 0.001	3.2 (2.0–5.0)	< 0.001
Age 75 years or older	1.8 (1.2–2.9)	0.009	1.9 (1.2–3.1)	0.006
Stage 3 or 4	1.5 (0.9–2.4)	0.088	-	

* The multivariate model is for 599 patients with complete data on all variables included in it.

** The multivariate model is for 600 patients with complete data on all variables included in it.

# Odds ratio

@ Confidence interval

### Response and survival

There was no statistically significant difference in either response rates or survival for patients with or without B symptoms.

## Discussion

These data demonstrate for the first time and in a large patient cohort that the presence of inflammatory (B) symptoms is an independent predictor of increased incidence of severe leucopenia and anaemia in patients with malignancy, in this case high risk, intermediate grade non-Hodgkin lymphoma. Although B symptoms were associated with an increased risk of thrombocytopenia, nausea and vomiting and diarrhoea on univariate analysis, these associations were not supported on multivariate analysis.

The study has some limitations. Firstly, it was an unplanned reanalysis of prospectively collected data, and the presence of inflammation was only assessed clinically as correlative blood samples for measurement of inflammatory proteins were not available. In addition, it was not possible to assess the impact of B symptoms on the incidence of neutropenia, febrile neutropenia, toxicity related hospitalisation, treatment related death and dose delay or reduction, as these data had not been reliably recorded. Such associations and correlation with plasma inflammatory markers should be evaluated in future studies. However, an 18% increase (40% vs 58%) in the incidence of grades 3 and 4 leucopenia, which was worse in patients with inflammatory symptoms is highly clinically significant and likely to impact on such outcomes.

This study was principally undertaken to provide a clinical "proof of principle" of our previous pre-clinical and clinical findings that the presence of an inflammatory response predicts for slower hepatic clearance of cytotoxic drugs and increased toxicity. The data will not have a major immediate impact on the management of patients with NHL, as prophylactic G-CSF is already routinely used in this condition, and our data confirm that this provides a protective effect against leucopenia. Also, in this cohort, the presence of inflammatory symptoms did not adversely impact on survival, which again raises questions about the immediate clinical significance of our findings. However, this may have been due in part to our desire to include only patients on whom full data sets were available for the assessment of toxicity and could have excluded patients who experienced early disease progression. Furthermore, multiple previous investigators have established that B symptoms are associated with worse progression free and overall survival in NHL [6-10].

As discussed above, B symptoms are indicators of a systemic inflammatory response and have been associated with elevated plasma concentrations of CRP and pro-inflammatory cytokines [6,9,10]. Moreover, clinical studies with anti IL-6 monoclonal antibody in patients with lymphoproliferative conditions resulted in reductions in CRP concentrations and improved clinical outcome [17]. There is substantial pre-clinical and clinical evidence from cancer and other inflammatory diseases, that hepatic drug clearance is reduced in the presence of a systemic inflammatory response [18-20]. A number of agents used in the management of NHL are metabolized by the liver including doxorubicin, cyclophosphamide and vincristine. As over 50% of all prescribed medications, including many cytotoxic agents, are metabolized by CYP3A4, this may be a significant source of pharmacokinetic and toxicity variability to anticancer agents [13,21-24].

The effects of cancer on hepatic drug metabolism have been recently clarified. Rivory and colleagues demonstrated that cancer patients with elevated acute phase proteins, including CRP and alpha-1 acid glycoprotein (AAGP), had reduced CYP3A4 function as determined by the erythromycin breath test [14]. The elevated plasma CRP concentrations were associated with increased plasma concentrations of IL-6. In addition, elevated levels of CRP were associated with reduced plasma clearance and increased toxicity following treatment with docetaxel, a CYP3A4 substrate [15]. By utilising a regulatory transgenic reporter mouse model of human CYP3A4, Charles and colleagues demonstrated that the presence of tumour resulted in down-regulation of the CYP3A4, which correlated with a systemic acute phase response. Furthermore, IL-6 expression was localised within the tumour, suggesting that the tumour or its associated stroma was the source of the increased circulating IL-6 noted in this model. As a result of this study a mechanistic link was suggested between tumour-derived cytokines and impaired drug metabolism [25]. Furthermore, in data from the same models, Sharma and colleagues demonstrated significant reductions in the expression of a number of drug transporters in the livers of tumour bearing mice compared to control animals, suggesting a global reduction in hepatic drug handling in the presence of malignancy.

These data make it likely that cytokines responsible for the B symptoms in NHL are also causing reduced hepatic drug clearance, resulting in the increased toxicity documented in the current study. This finding has a number of important clinical and research implications for NHL and cancer in general. Firstly, it means that we have a useful marker for increased toxicity in NHL. Although a number of prognostic indices have been used in NHL most have been used to predict for response and survival rather than toxicity. The ability to identify patients at increased risk of myelosuppression and infection will permit increased surveillance and possibly increased use of prophylactic antibiotics. Furthermore, the prognostic utility of B symptoms could be enhanced by measuring plasma proteins including CRP or cytokines such as IL-6. The availability of multiplex bead cytokine analyses that permit simultaneous measurement of multiple cytokines should facilitate such research. These findings may also be applicable to other malignancies where it is likely that inflammation is responsible for a significant proportion of the variability in toxicity from chemotherapy. However, as overall survival has consistently been lower in patients with elevated inflammatory markers [7-10] it would be potentially hazardous to manage an increased risk of toxicity by dose reduction as this could further compromise response and survival. It may also be possible to use inflammatory markers to more appropriately guide the use of colony stimulating factors in patients with solid tumours.

In addition to providing prognostic information, the inflammatory proteins provide a potential target for novel therapies aimed at reversing the inflammatory effects, improving drug handling and reducing toxicity. A number of antibodies have been developed to target pro-inflammatory cytokines, including TNF-α, IL-6 and IL-6 receptor, and these should be investigated for their potential to reverse tumour-associated impairment of drug metabolism. There are preliminary data to suggest that this approach might also help to overcome tumour resistance. However, these data require confirmation in prospective clinical studies that also involve the collection of plasma for measurement of acute phase proteins and cytokines.

## Conclusion

This study shows that patients with aggressive non-Hodgkin lymphoma who have B symptoms experience significantly more myelosuppression than those without inflammatory symptoms. The toxicities were less in patients who had received G-CSF. These data support the hypothesis that patients with a cancer induced inflammatory reaction have reduced hepatic metabolism of cytotoxic drugs which results in the increased toxicity. These results might help explain some of the substantial inter-patient differences in toxicity from chemotherapy.

## Competing interests

The authors declare that they have no competing interests.

## Authors' contributions

RS carried out liaison with the BNLI to obtain the dataset for the manuscript and was responsible for preparing an initial version of the manuscript. PS and DC were responsible for providing the UK side of the BNLI liaison and for providing data, answering data queries and reviewing the manuscript. OD was responsible for the statistical analysis and providing input into the later versions of the manuscript. GR and SC initiated and jointly supervised the project. All authors read and approved the final manuscript.

## Pre-publication history

The pre-publication history for this paper can be accessed here:

http://www.biomedcentral.com/1471-2407/9/153/prepub
